# Determination of selected gadolinium-based contrast agents in soil: method validation and application

**DOI:** 10.1007/s00216-026-06389-2

**Published:** 2026-02-11

**Authors:** A. F. Roig-Navarro, F. Soria-Prieto, E. Pitarch, R. García-Cubedo

**Affiliations:** Environmental and Public Health Analytical Chemistry, Research Institute for Pesticides and Water, Universitat Jaume I, Avgda. Sos Baynat, S/N, 12071 Castelló, Spain

**Keywords:** Gadoteric acid, Gadobutrol, Gadoteridol, Soil, IC-ICP-MS

## Abstract

The increasing use of reclaimed water for agricultural irrigation raises concerns about the environmental accumulation of persistent contaminants, including gadolinium-based contrast agents (GBCAs). These compounds, widely used in medical imaging, are excreted unmetabolized and poorly removed by conventional wastewater treatment plants, leading to their release into aquatic environments and potential accumulation in soils and crops. In this study, a method was developed, validated, and applied—for the first time—for the determination of three commonly used GBCAs—gadoteric acid, gadoteridol, and gadobutrol—in clay soil. Quantification was achieved by ion chromatography coupled to inductively coupled plasma mass spectrometry (IC-ICP-MS). Suitable separation was accurately optimized using a mobile phase containing 30 mM EDTA and 20 mM NH_4_NO_3_ at pH 9.8. Elution of all target species was achieved within 7.5 min. The method was evaluated using real clay soil samples from an agricultural field in Castelló (Spain) fortified with selected GBCAs at various concentrations. Mild extraction with NH_4_NO_3_ yielded recoveries below 50%, which allows assessment of the bioavailable fraction. To achieve quantitative recovery, a more severe alkaline extraction protocol using KOH was subsequently developed. The optimized procedure demonstrated excellent analytical performance, with LOD and LOQ below 2 and 5 ng g^−1^, respectively. This methodology enabled the first quantitative determination of GBCAs in soil samples collected from municipal parks irrigated with reclaimed water. Furthermore, the KOH-based extraction was extended to the quantification of gadoteric acid in peat substrates from greenhouse-scale cultivation trials, yielding a mean recovery of 80% with a relative standard deviation (RSD) of 12%. The application of this method confirmed the accumulation of the contrast agent in peat irrigated with fortified tap water. These findings underscore the necessity for robust, matrix-specific analytical workflows to monitor the environmental fate of GBCAs and to accurately assess their potential transfer into the food chain.

**Graphical abstract:**

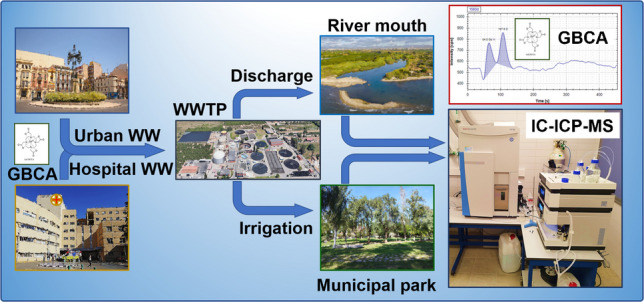

**Supplementary Information:**

The online version contains supplementary material available at 10.1007/s00216-026-06389-2.

## Introduction

Water scarcity is a significant global challenge, driven by factors such as population growth and climate change. In arid and semi-arid regions, limited freshwater resources cannot meet increasing demands, leading to groundwater over-extraction and environmental degradation, which threaten food production and ecosystem health [1, 2]. In a context where agriculture is the leading consumer of freshwater, reclaimed water has emerged as an alternative for crop irrigation. It offers a sustainable solution to alleviate pressure on freshwater supplies by recycling water. In addition, its reuse contributes to safeguarding natural ecosystems from pollution [2–8]. Nevertheless, the effective elimination of persistent contaminants from treated wastewater is imperative; alternatively, their concentrations must be rigorously monitored and maintained within acceptable thresholds [6–8]. The need for reliable analytical data on the long-term impact of those contaminants is clear.

Recently, rare earth elements (REEs), classified as critical technological elements due to their growing use in specialized applications, have been incorporated into the group of target analytes in treated water and sludge [9]. Among them, gadolinium, a metal of medical use, is gaining relevance. Gadolinium-based contrast agents (GBCAs) are commonly used in magnetic resonance imaging (MRI) to enhance the visibility of soft tissues. Those compounds have raised environmental concerns, particularly in aquatic ecosystems, due to their persistence and potential toxicity. After being administered, GBCAs are excreted within 24 h by patients, primarily through urine, and eventually make their way into sewage systems reaching wastewater treatment plants (WWTP). However, conventional WWTP are not designed to remove or degrade these complexed forms of gadolinium. As a result, GBCAs are discharged into rivers, lakes, and coastal waters, where they accumulate over time. These compounds are known for their exceptional thermodynamic and kinetic stability, resulting in environmental persistence and long half-lives [10, 11]. Stability assessments under varying physicochemical conditions have shown that these complexes remain largely intact at elevated temperatures, across slightly acidic to alkaline pH ranges, and under UV or solar irradiation [12, 13]. Thus, significantly elevated gadolinium concentrations—relative to natural geochemical background levels—have been detected in environmental water near urban areas with advanced health systems with high MRI usage [10, 14–17]. Consequently, speciation analysis of Gd—rather than total gadolinium quantification—is essential to accurately characterize anthropogenic Gd anomalies within aquatic and terrestrial ecosystems. Indeed, these complexes have been detected in tap water [18, 19] across several European cities and even in water-based beverages [19], indicating their entry into the human food chain and raising public health concerns [20]. Therefore, these compounds should be systematically monitored in affected aquatic environments.

The determination of GBCAs in diverse matrices mainly employs liquid chromatography coupled with inductively coupled plasma mass spectrometry (LC-ICP-MS). Most reported methods for water analysis utilize hydrophilic interaction liquid chromatography (HILIC) as the separation mechanism [21–25]. To overcome challenges related to the introduction of high organic solvent content into the plasma during GBCA analysis, anion exchange chromatography (IC) has recently been proposed as a suitable alternative [26–29]. This approach eliminates the need for platinum cones in ICP-MS and avoids the requirement for additional oxygen flow, thereby simplifying instrumental conditions.

Current analytical research has primarily focused on the determination of GBCAs in aqueous matrices [10, 14–17], while speciation analysis in solid samples remains largely unexplored. Consequently, knowledge regarding the bioavailability, mobility, and transformation of gadolinium species within biological systems—and their subsequent transfer into the terrestrial food chain—remains limited [11]. Solid matrices introduce additional analytical challenges, necessitating extraction strategies that preserve the native chemical form of gadolinium complexes. Schlatt et al. [30] developed a species-specific quantification method successfully applied to rat femurs following GBCA administration. Similarly, Lindner et al. [31] examined contrast agent uptake in cress plants, and Sommer et al. [32] employed single-cell ICP-MS to evaluate GBCA accumulation in algae. Moreover, Scurtu et al. [33] reported the bioaccumulation of gadobutrol in Stevia rebaudiana. However, the last three approaches primarily determine total gadolinium concentrations rather than intact GBCA species. Regarding terrestrial matrices, laboratory-scale investigations by Menahem et al. [34]—representing a singular study on GBCA transport in soil—demonstrated the high mobility of gadopentetic acid (a linear GBCA). Furthermore, Sommer et al. [35] indicated that macrocyclic GBCAs exhibit negligible interaction with humic acids. To date, however, no studies have achieved the direct quantification of intact GBCAs in environmental soil samples. Considering that plant uptake from the soil is a pivotal pathway for entry into the terrestrial food chain, such investigations are essential to elucidate environmental exposure routes [11, 33], particularly in the context of irrigation with reclaimed wastewater [6–8].

This study is part of a broader research initiative evaluating the reuse of reclaimed water for agricultural irrigation. The primary objective is to develop, validate, and apply an IC-ICP-MS method for the determination of selected GBCAs in soil. Method validation was conducted using authentic clay soil samples from an agricultural field and subsequently applied to soil samples from municipal parks irrigated with reclaimed water. A mild NH_4_NO_3_ extraction allowed to assess the bioavailable fraction, and a more rigorous KOH-based protocol was used for the determination of total GBCA concentrations. Additionally, as part of the broader research on reclaimed water reuse, the alkaline extraction procedure was evaluated for gadoteric acid using peat samples from greenhouse experiments. To the best of our knowledge, this work represents the first study dedicated to the quantification of intact GBCAs in soil matrices. The target analytes—gadoteric acid, gadobutrol, and gadoteridol—were selected to reflect current clinical usage in the Castelló province (Spain). This selection aligns with the European Medicines Agency (EMA) regulatory framework, which has restricted the use of certain linear GBCAs since 2017 in favour of more stable macrocyclic complexes [36–38]. This shift in clinical preference is further corroborated by recent environmental monitoring data, such as the predominance of macrocyclic species in the Ruhr River [29].

## Materials and methods

### Standards and reagents

Gadoteric acid (Dotarem) (Gd-DOTA, 0.5 mmol mL^−1^. Guerbet, Shirley, UK), gadoteridol (Gd-HP-DO3A, 0.5 mmol mL^−1^. BRACCO IMAGING S.p.A. Colleretto Giacosa (TO) Italy), and gadobutrol (Gd-BT-DO3A, 1 mmol mL^−1^. Bayer, Berlin, Germany) were provided by the “Hospital General Universitari de Castelló” (Castelló, Spain). Stock solutions with a nominal concentration of 500 mg L^−1^ (as Gd) were prepared from the original drug vials by dilution with deionized water (DW). Stock solutions for each compound were properly diluted to 1 ng mL^−1^ (as Gd) with DW. Using these diluted solutions, the purity of each compound was tested with IC-ICP-MS (only one chromatographic peak was detected) and the exact concentration checked against a calibrator prepared with inorganic Gd certified standard (Gd standard for ICP, Scharlab, Barcelona, Spain) by ICP-MS. Mixed intermediate solutions of 50 mg L^−1^ (as Gd) were prepared by diluting the stock solutions with DW. Calibration solutions were prepared daily by appropriate step dilutions of intermediate solutions with DW.

HNO_3_ acid (trace metal basis) and KOH (analytical reagent) were purchased from Merck (Darmstadt, Germany). NH_4_NO_3_ (analytical reagent) was from Sigma-Aldrich (Darmstadt, Germany), EDTA acid complexing agent (synthesis grade) was from Scharlab (Barcelona, Spain), and NH_4_OH (25%. Analytical reagent) was from Supelco (Darmstadt, Germany). DW (resistivity, 18.2 MΩ cm) was obtained from a Ultramatic GR (Wasserlab, Barbatáin (Navarra), Spain). Methanol (MeOH, LC-MS grade) was acquired from Scharlab.

### Instrumentation

The analysis of GBCAs was carried out using an IC-ICP-MS coupled system. The separation was conducted by a Dionex Ultimate 3000 LC system using a Hamilton (Reno, NV, USA) PRP-X110 anion exchange column (150 × 1 mm, 7 µm). The LC system was connected to an iCAP RQ ICP-MS Thermo Fisher Scientific (Bremen, Germany). Signal was registered at *m/z* 158 corresponding to Gd. ICP-MS operating conditions were optimized daily to maximize sensitivity while minimizing double charged ions and polyatomic interferences. The measurements were conducted using a collision cell in He mode (daily) optimized at (nearly) 4.35 mL min^−1^. A dwell time of 200 ms was utilized. The chromatographic data were processed using the instrument's native software, applying a “moving mean” smoothing algorithm. All plastic and glass labware were rinsed in a 10% HNO_3_ bath for 24 h before use. The finally optimized separation method was conducted using a flow (0.2 mL min^−1^) of a mobile phase based on 30 mM EDTA and 20 mM NH_4_NO_3_ at pH = 9.8. A step gradient of MeOH was also applied (from 2 to 4 min a 3% MeOH concentration was added to mobile phase). Gd^3+^ and the three selected contrast agents were satisfactorily separated in 7.5 min. A 6-point calibration curve (including a blank) was prepared in DW.

### Validation procedure

For validation purposes, the typical clay soil sample was collected from an agricultural field near Castelló City. Sampling was carried out using a shovel to obtain a surface soil sample of about 1 kg. The soil sample was spread in a fume hood, air-dried for at least 72 h, homogenized by gentle crushing, and subsequently sieved. The particle fraction smaller than 0.5 mm was selected for fortification and extraction experiments (the mesh was selected specifically to maximize sample homogeneity for the trace-level detection of GBCAs). Peat, provided by the greenhouse facilities from Universitat Jaume I, was acquired from Free Peat B.V (Vriezenveen, The Netherlands). An excess of endogenous free Gd^3+^ was detected in the peat matrix, leading to compromised recoveries during method validation with fortified samples. To mitigate interference from native Gd^3+^, the peat matrix was treated with EDTA to effectively remove the Gd^3+^ species (see details in Supplementary Information). All subsequent validation experiments were performed using the EDTA-treated (stripped) peat matrix. Both materials, the native clay soil and the commercial peat, were confirmed to be free of detectable GBCAs prior to fortification and extraction experiments.

To assess method precision and trueness, dry samples were spiked with the selected compounds (gadoteric acid, gadobutrol, and gadoteridol) at two mass fraction levels: 5 and 25 ng g^−1^ for clay soil, and 10 and 50 ng g^−1^ for the stripped peat matrix (while gadoteric acid was the primary analyte of interest for the peat substrate, gadobutrol and gadoteridol were also incorporated into the validation protocol to evaluate the method’s applicability for multi-residue analysis in this matrix). For each level, 100 g of material was spiked using 50 mL of aqueous standard solution (sufficient to achieve complete wetting). The mixtures were thoroughly homogenized to ensure uniform contact between the solid and liquid phases and subsequently air-dried in a fume hood for more than 48 h. This procedure was intended to simulate the adsorption processes occurring during irrigation with reclaimed water and subsequent drying of the soil under field conditions.

Based on the chemical stability and high mobility of GBCAs [10–17, 34, 35], a mild extraction procedure using NH_4_NO_3_ was initially evaluated—at 25 ng g^−1^ fortification level—to determine the operationally defined bioavailable fraction. For these experiments, 1 g of soil was treated with 10 mL of NH_4_NO_3_ solution. Method optimization involved varying the pH (0.5–12), extraction time (15–60 min), and extractant concentration (5 mM and 200 mM). Specifically, a 200 mM NH_4_NO_3_ solution at pH 9.2 with a 30 min contact time was also investigated. All experiments were performed in triplicate.

To achieve quantitative recovery of the total GBCA concentration, an alkaline extraction method was developed. Given the high polarity and low molecular weight of GBCAs, an existing protocol for glyphosate extraction from soil [39] was adapted and optimized. For the final procedure, 1 g of soil was extracted with 15 mL of 0.5 M KOH. To ensure representative sampling of the more heterogeneous peat matrix, 2 g of peat were extracted with 30 mL of 0.1 M KOH. The analytical performance of the KOH-based method was rigorously validated. Intra-day precision was evaluated using five replicates at each concentration level. Inter-day precision was assessed over four non-consecutive days using 11 replicates per level. Trueness was determined through recovery studies of fortified samples.

In addition, single extraction was compared with two consecutive extraction steps using the same solid/liquid ratio for both extraction procedures.

All extractions were performed using a rotary agitator at 50 rpm for 30 min. Following extraction, the suspensions were centrifuged at 6000 rpm for 10 min. The resulting supernatants were filtered through 0.22 µm nylon membranes and diluted 1:10 (v/v) with deionized water prior to IC-ICP-MS analysis.

Compound stability under the strong alkaline extraction conditions was evaluated by comparing the analytical responses of aqueous standards to those of standards subjected to the complete KOH procedure at equivalent concentrations. No statistically significant differences were observed, confirming the integrity of the GBCAs throughout the extraction process. Furthermore, potential matrix effects were investigated by comparing aqueous standards with matrix-matched standards prepared from blank soil and peat extracts. These experiments were conducted at two concentration levels—near the LOQ and at 5× LOQ—and demonstrated the absence of significant signal suppression or enhancement. Additionally, the consistency of retention times was verified by comparing aqueous standards with fortified blank matrix extracts, showing no chromatographic shifts. In summary, the optimized method exhibited high operational stability for all target analytes and a negligible matrix effect, allowing for quantification using external calibration.

### Application to real samples

The validated analytical method was applied to several real samples irrigated with reclaimed water (clay soil) and tap water spiked with gadoteric acid (peat):

- Eight randomly selected samples from two municipal parks in Castelló were collected. Around 0.5 kg of surface soil was collected, left to dry, crushed, and sieved below 0.5 mm.
- Four peat samples were obtained from a greenhouse experiment using endive (Cichorium endivia) as the test crop. The study, performed in triplicate, involved plants grown in 2 L pots and irrigated with water fortified with gadoteric acid at four concentration levels: 0.5, 5, 50, and 500 µg L⁻^1^. Irrigation was carried out twice weekly with 200 mL of the respective solution over an 8-week period. At the end of the cultivation, approximately 10 g of representative pooled peat was collected for each irrigation level and subsequently air-dried.

## Results and discussion

### Separation optimisation

As an initial approach, the IC separation method developed by Macke et al. [27], which employs a step gradient of NH_4_NO_3_ (14 to 125 mM) at pH 9.2 using an anion exchange column (CF-Gd-01, 50 × 4 mm, Elemental Scientific Inc, Omaha, NE, USA) was evaluated. Although this column performed well for water samples, it failed to achieve separation of GBCAs when KOH soil or peat extracts were injected. The high eluent strength of the sample matrix prevented GBCA retention on the column. Conversely, the mobile phase gradient did not yield satisfactory separation with the Hamilton PRP-X110 anion exchange column (150 × 1 mm), which was ultimately employed in the present study. Furthermore, increasing the NH_4_NO_3_ concentration to 200 mM resulted in nebulizer clogging after prolonged operation. Consequently, a detailed investigation of the mobile phase composition, based on NH_4_NO_3_, EDTA, and MeOH, as well as the pH, was carried out.

Increasing the EDTA concentration facilitated the elution of inorganic Gd^3^⁺—as the Gd-EDTA complex—near the column dead volume, achieving baseline resolution from gadoteric acid (D), which eluted subsequently. In contrast, increasing the NH_4_NO_3_ concentration primarily reduced the retention time of gadobutrol (GB), while the presence of a low percentage of MeOH particularly affected gadoteridol (GT) retention. Although the underlying mechanism remains unclear, the stronger retention of gadoteridol suggests enhanced hydrophobic interactions with the polymeric-based stationary phase. The addition of a small proportion of MeOH effectively minimised these interactions. Further pH optimization showed that maximum resolution was obtained at pH 9.8. Although inorganic Gd^3^⁺ was not specifically investigated in the present study, its effective separation from GBCAs must be ensured to avoid interferences and inaccuracies in quantification.

**Fig. 1:**
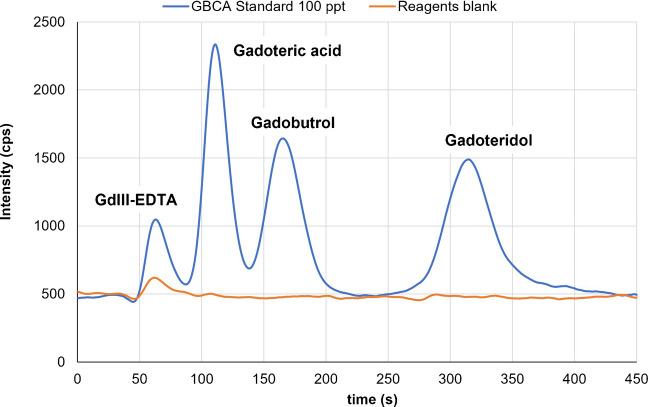
Chromatogram showing the achieved separation of GBCA with the PRP-X110 anion exchange column at 0.2 mL min^−1^ (see text for mobile phase details). D, GB, and GT nominal concentration was 100 ng L^−1^. GdIII-EDTA complex concentration was 25 ng L^−1^

The final optimized separation conditions consisted of a mobile phase containing 20 mM NH_4_NO_3_, 30 mM EDTA, and a MeOH step gradient (from 0 to 2 min, 0%; from 2.1 to 4 min, 3%; from 3.1 to 7.5 min, 0%) adjusted to pH 9.8. Under these conditions, all Gd-based compounds—including GdIII-EDTA complex—were properly separated within 7.5 min (Fig. 1). An injection volume of 20 µL and a flow rate of 0.2 mL min^−1^ were used.

### Extraction optimisation

In view of the high chemical stability and environmental mobility of GBCAs, various mild extraction conditions using NH_4_NO_3_ solutions were evaluated to recover these analytes from fortified soil samples (25 ng g^−1^). Parameters including pH, extraction time, and extractant concentration were systematically investigated. Stability and the absence of matrix effects were confirmed using fortified blank reagents and soil extracts, respectively. As summarized in Table 1, all tested conditions yielded mean recoveries ranging from 20% for GB to 50% for D and GT, with relative standard deviations (RSDs, *n* = 3) below 15%. The implementation of successive extraction cycles yielded a marginal improvement in cumulative recovery of less than 5%. Given this negligible enhancement in analyte yield, the extraction protocol was finalized as a single-step procedure, and further optimization was deemed unnecessary.

**Table 1:** Recovery of selected GBCAs from clay soil following extraction with NH_4_NO_3_ under various experimental conditions. Fortification level, 25 ng g^−1^

NH_4_NO_3_ conc	pH	Extr. time (min)	Recovery* (%)		
			Gadoteric acid	Gadobutrol	Gadoteridol
5 mM	0.5	15	44	31	40
		30	40	27	37
		60	37	21	33
	5.5	15	43	24	36
		30	38	22	34
		60	52	29	43
	9.2	15	42	22	35
		30	49	22	41
		60	46	24	40
	12	15	52	26	43
		30	49	25	42
		60	43	21	38
200 mM	9.2	30	43	30	46

*RSD (*n* = 3) value for any recovery was lower than 15%

The extraction efficiency of the NH_4_NO_3_ reagent is primarily attributed to ion exchange mechanisms at matrix interaction sites. This process facilitates the release of both the ionic species, such as D, and the high-polarity compounds, GB and GT, from the soil matrix. Consequently, the GBCAs recovered using this reagent represent the operationally defined bioavailable fraction. GB constantly exhibited lower recoveries, a trend attributed to its somewhat distinct molecular structure and slightly higher molecular weight (see Table S1, in Supplementary Information), which promotes more robust interactions with the clay soil matrix.

Given the environmental persistence of these macrocyclic complexes and their potential for entering the terrestrial food chain under varying environmental conditions, a more rigorous alkaline extraction using KOH was subsequently developed to achieve quantitative recovery of the target compounds.

As before, and according to the experimental section, the compounds’ stability and the absence of matrix effects were carefully examined. No such negative effects were found along the operational conditions used. Optimal recovery was obtained using 0.5 M KOH, while the addition of 25% MeOH did not improve yields for any of the GBCAs tested. The final optimized extraction procedure consisted of treating 1 g of spiked, dried soil with 15 mL of 0.5 M KOH, followed by end-over-end agitation (50 rpm, 30 min). The resulting suspension was centrifuged (6000 rpm, 10 min), filtered through a 0.22 µm nylon membrane, and diluted 1:10 to prevent matrix effects on both instrumental signal and chromatographic separation. In line with the above-shown extraction trials, the implementation of successive extraction cycles yielded only a marginal increase in cumulative recovery. Accordingly, a single-step extraction procedure ended the workflow, as further optimization offered no significant gains in yield for the intended application. The figures of merit of the optimized method, including validation parameters, are presented in the following section.

### Method validation: intra- and inter-day precision, LOQ, trueness

Following the procedure outlined in the “Validation procedure” section, the optimized method was validated through the evaluation of trueness and precision. Method intra-day precision was assessed using spiked soil samples at mass fraction levels of 5 and 25 ng g^−1^, each analysed in quintuplicate. Inter-day precision was determined from a total of 11 replicates obtained over four different days. Mean recovery values and relative standard deviations (RSDs) are summarized in Table 2. Limits of detection (LOD) and quantification (LOQ) were calculated as three and ten times the standard deviation (SD), respectively, where SD corresponds to the measured concentration variability from the repeatability experiments at the lowest spiking level.

**Table 2:** Mean recovery values and RSD (%), LODs and LOQs for the selected GBCA in clay soil samples fortified at two concentration levels

Mean recovery (RSD), %							
Fortification level	Intra-day precision (*n* = 5)				Inter-day precision (*n* = 11)		
	D		GB	GT	D	GB^a^	GT
5 ng g^−1^		81 (5)	48 (10)	90 (11)	82 (7)	49 (10)	89 (12)
25 ng g^−1^		87 (1)	61 (3)	93 (5)	84 (6)	59 (7)	88 (9)
Limits of detection and quantification							
LOD (ng g^−1^)		1	1	2			
LOQ (ng g^−1^)		2	4	5			

^a^To account for the systematic bias arising from the low recovery of GB, the expanded measurement uncertainty (U, k = 2) was evaluated. In accordance with the Eurachem/CITAC Guide [40], a conservative estimate at a mass fraction of 5 ng g^−1^ yielded a relative expanded uncertainty of 20%

Trueness in the clay soil matrix, evaluated through recovery experiments, was satisfactory for D and GT, with mean recoveries close to 90% at both tested mass fractions. GB showed comparatively lower recoveries (≈50% or slightly higher). RSD values for all analytes were below 12%, confirming acceptable method precision. Given this acceptable precision, GB concentrations in real-world samples can be reported after correction for recovery bias. To quantify the impact of this correction on the reliability of the results, the combined measurement uncertainty must be estimated in case of positive samples. (See Table 2 for the assessment of that impact in GB recovery uncertainty). Overall, the method was considered adequately validated for the clay soil matrix and suitable for the determination of the selected GBCAs in such samples.

As anticipated, the alkaline extraction protocol yielded significantly higher recoveries compared to the NH_4_NO_3_ treatment. This increased efficiency is attributed to the ability of the KOH solution to partially solubilize the silicate matrix and the soil organic matter, both of which serve as the primary sorption phases for GBCAs. By degrading these reactive components, the analytes are more effectively released into the liquid phase.

Notably, GB consistently demonstrated lower extraction efficiencies relative to the other target analytes, mirroring the trend observed during the mild extraction experiments. This persistent behaviour across both extraction regimes further corroborates that the specific molecular configuration and higher molecular weight of GB favour more robust interactions with the clay soil components, which continue to hinder its extractability even under rigorous alkaline conditions.

LODs and LOQs of ≤ 2 and ≤ 5 ng g^−1^, respectively, were achieved for all three GBCAs. Specifically, for D—the most widely used GBCA in the Castelló province healthcare system and consistently the predominant species in local environmental waters (see Table S2, Supplementary Information)—LOD and LOQ values were 1 and 2 ng g^−1^, respectively. These sensitivity levels enabled the detection of D in soil samples from municipal parks irrigated with reclaimed water (see the “Application to real samples” section).

As part of the broader research on reclaimed water reuse for agricultural irrigation, the extraction procedure previously validated for clay soil was adapted for the peat matrix used in greenhouse experiments (see “validation procedure” section). Gadoteric acid was the only organometallic species targeted for the peat-based studies (along with several organic micropollutants); however, the method’s intention was extended to include all three selected GBCAs to evaluate the procedure’s broader applicability. For this matrix, initial trials using 0.5 M KOH resulted in the formation of a stable colloidal suspension, leading to immediate clogging of the 0.22 µm filtration membranes, making the sample preparation process unsuitable for routine analysis even after a pre-filtration and centrifugation steps. The transition to 0.1 M KOH optimized the analytical workflow by enhancing extract filtration while maintaining satisfactory recoveries for D. Thus, a 0.1 M KOH extracting solution was finally chosen, while all other extraction parameters remained consistent with the original method. The adapted procedure was validated using stripped peat samples spiked at 10 and 50 ng g^−1^.

Initial recovery experiments yielded unacceptably high values for D (150%), along with poor reproducibility, as indicated by RSD values occasionally exceeding 50% (results not shown).

While no matrix effects were observed in KOH extracts and no GBCAs were detected in the native peat, further investigation identified endogenous Gd^3+^ at ng g^−1^ levels. Notably, this Gd^3+^ species was only detectable following an EDTA extraction and remained undetected when the non-spiked peat was extracted with KOH. This indicates that the endogenous gadolinium exists in a form that can only be mobilized by a strong chelating agent. The resulting chromatograms showed an excessively broad and poorly reproducible Gd-EDTA peak that overlapped with the retention times of D and, in some instances, GB, thereby compromising specificity and trueness as shown in Figure S2 of the Supplementary Information. Consequently, the peat matrix was pre-treated with EDTA to strip the endogenous Gd^3+^ as described in the section. Following this treatment, blank chromatograms exhibited only a minimal signal (Figure S2), and validation of the EDTA-treated matrix yielded satisfactory results for D, with recoveries above 80% and RSDs below 12% at both concentration levels (Table 3). Although EDTA treatment may modify the native peat structure, it was assumed that such modifications do not significantly alter the KOH extraction efficiency for GBCAs in real samples.

**Table 3:** Mean recovery values and RSD (%), LODs and LOQs for the selected GBCA in peat samples fortified at two concentration levels

Mean recovery (RSD) %						
Fortification level	Intra-day precision (*n* = 5)			Inter-day precision (*n* = 11)		
	D	GB	GT	D	GB	GT
10 ng g^−1^	85 (4)	40 (6)	16 (18)	87 (7)	39 (7)	22 (7)
50 ng g^−1^	82 (9)	37 (7)	18 (7)	90 (12)	38 (7)	20 (13)
Limits of detection and quantification						
LOQ (ng g^−1^)	4	6	14			
LOD (ng g^−1^)	1	2	5			

Regarding GB and GT, recovery values were notably lower, at approximately 40% and 20%, respectively, indicating that the extraction procedure requires further optimization for these specific compounds in the peat matrix. The attenuated extraction efficiencies observed for GB and GT are likely attributable to their non-ionic character, which facilitates stronger hydrophobic interactions with the organic-rich peat matrix. Despite these lower recoveries, the method demonstrated acceptable precision, with RSD values consistently below 7% for GB and between 7 and 18% for GT. Consequently, alternative extraction strategies employing reagents with enhanced elution strength are currently under investigation to improve the recovery of these neutral species. Nonetheless, the application of the current method to real peat samples from the greenhouse experiment was restricted to the determination of D.

For gadoteric acid, the LOD and LOQ were 1 and 4 ng g^−1^, respectively, both well below the concentrations typically detected in real peat samples (see the “Application to real samples” section below), and thus suitable for the intended analytical objectives.

The operational stability demonstrated by GB and GT during optimization suggests that the lower yields are not a consequence of chemical degradation. Instead, the differential extractability observed across the analyzed matrices may be linked to the distinct physicochemical properties of the complexes under the alkaline conditions of the KOH extraction (pH ≈ 12). At this pH, GB and GT are characterized as neutral species, whereas D remains anionic [10, 14–16, 41]. The alkaline medium partially solubilizes the matrix components of both the clay and peat while inducing a negative charge on the silicate and organic matter constituents. In this environment, the anionic D might experience a degree of electrostatic repulsion from the matrix surface, a mechanism that could facilitate its release into the extractant and account for its consistently higher recovery. Conversely, the neutral character ascribed to GB and GT suggests they may be more susceptible to non-specific hydrophobic interactions or partitioning within the organic fraction. The absence of electrostatic repulsion for these neutral macrocycles might allow for stronger retention within the substrate—particularly in the organic-rich peat—potentially leading to the attenuated recoveries observed. While the addition of MeOH as an organic modifier did not enhance yields, further refinement of the extraction protocol for GB and GT in peat is currently being explored.

### Application to real samples

**Fig. 2:**
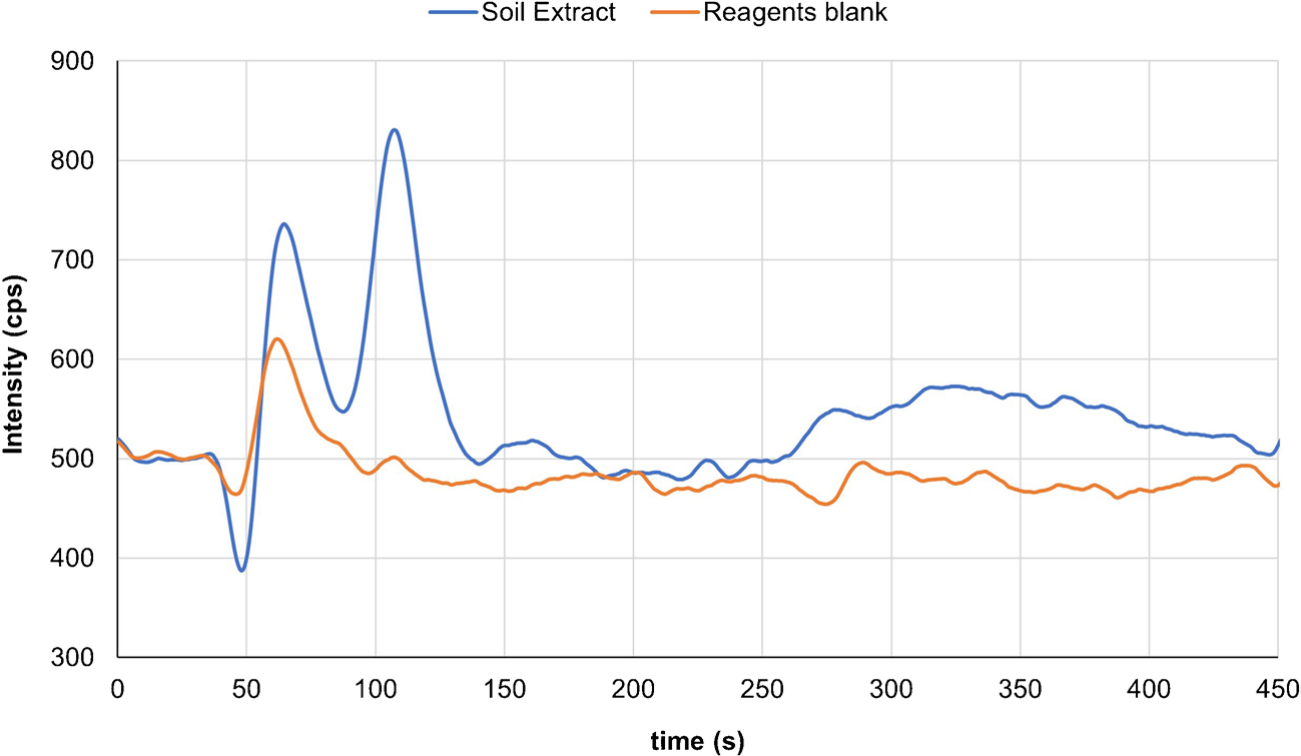
The chromatogram corresponding to the P. Rafalafena_C selected clay soil sample is shown. Gd-EDTA complex (not quantified) and D at 12 ng L^−1^ in the extract (1.4 ng g^−1^ in soil) were detected. A clear peak near the LOD for D was resolved from the baseline

The validated method was applied to eight clay soil samples collected at random from two municipal parks in Castelló (Spain) (Figure S.1, Supplementary Information). As shown in Table 4, among the GBCAs selected, only D was detected in the samples analysed, supporting again its high use in Castelló province healthcare. Six of the seven samples tested positive for D were found at concentrations falling between the LOD and LOQ established for this compound. Figure 2 depicts the chromatogram obtained for “P. Rafalafena_C” selected sample, where a distinct chromatographic peak, corresponding to a mass fraction close to the LOD for D, was clearly resolved from the baseline. Only one sample (P Rafalafena_B) yielded a quantifiable mass fraction of 3.2 ng g^−1^. These results indicated that irrigation with reclaimed water leads to the accumulation of D in soils subjected to such practices. This finding highlights the necessity of monitoring the composition of reclaimed water for the presence of GBCAs and supports the inclusion of these compounds in the group of persistent, mobile, and toxic substances to be routinely tracked in irrigated soils and environmental matrices. Furthermore, additional research is required to investigate the presence of GBCAs in crops cultivated in these soils, to properly evaluate their potential entry into the food chain.

**Table 4:** Concentration of gadoteric acid (D) in several clay soil and peat samples

Concentration of gadoteric acid as Gd, ng g^−1^	
Soil of municipal parks irrigated with Castelló WWTP effluent water^a^	[D] ± U^b^, ng g^−1^
P. Litoral_A	1.0* ± 0.1
P. Litoral_B	nd
P. Litoral_C	1.0* ± 0.1
P. Litoral_D	1.3* ± 0.2
P. Rafalafena_A	1.0* ± 0.1
P. Rafalafena_B	3.2 ± 0.4
P. Rafalafena_C	1.4* ± 0.2
P. Rafalafena_D	1.6* ± 0.2
Peat from greenhouse irrigated with fortified water	[D] ng g^−1^
Irrigation at 0.5 µg L^−1^	1.7* ± 0.2
Irrigation at 5 µg L^−1^	12 ± 2
Irrigation at 50 µg L^−1^	1722 ± 237
Irrigation at 500 µg L^−1^	9526 ± 1312

^a^MEAN concentration of D (as Gd) in effluent wastewater sample: 276 ng L^−1^ (See Table S2, Supplementary Information)

^b^U: expanded uncertainty (*k* = 2) calculated from inter-day recovery validation data according to EURACHEM/CITAC Guide [40]

*Mass fraction values between LOD and LOQ

The method, successfully validated for D in peat samples, was applied to four samples obtained from an experimental study conducted under controlled greenhouse conditions. In this study, crops were irrigated with water containing four different concentrations of D. As presented in Table 4, the results again confirmed the accumulation of D in the substrate (peat) where the crops were cultivated after 8 weeks of watering. As can be seen, only one sample (corresponding to irrigation at the lowest level) was detected at a mass fraction below its LOQ (4 ng g^−1^). Moreover, a clear positive correlation was observed between the concentration of D in the irrigation water and its accumulation in the peat matrix.

All these findings further underscore the need to develop and apply robust analytical methodologies for the determination of GBCAs in edible plants grown in substrates irrigated with reclaimed water, to accurately assess their potential entry into the food chain.

## Conclusions

A novel analytical method based on IC-ICP-MS was developed for the determination of selected GBCAs—D, GB, and GT—in clay soil and D in peat matrices. The use of an aqueous mobile phase effectively eliminated issues commonly associated with high organic solvent content, such as carbon deposition on ICP-MS interface cones and plasma instability, which are frequent in HILIC-based separations. Optimal chromatographic performance was achieved using a mobile phase containing 30 mM EDTA and 20 mM NH_4_NO_3_ at pH 9.8, delivered at 0.2 mL min^−1^. Suitable resolution was obtained for free Gd^3+^ (as the Gd-EDTA complex), D, GB, and GT within 7.5 min. In the absence of certified reference materials, method validation was performed via fortification experiments at two mass fraction levels.

Given the chemical stability and environmental mobility of GBCAs, mild extraction using NH_4_NO_3_ solutions was initially employed to assess the bioavailable fraction in the clay soil matrix. Recoveries for fortified spiked samples remained below 50% for all compounds, likely due to the limited extraction capacity of the ion exchange mechanism associated with NH_4_NO_3_. In contrast, a more rigorous alkaline extraction using KOH, which partially dissolves the silicate and organic matter matrix, yielded quantitative recoveries and high precision for all analytes. The stability of GBCAs and the absence of matrix effects during the procedure were confirmed through the analysis of fortified blanks and soil extracts. With limits of detection and quantification below 1 and 2 ng g^−1^, respectively, the method enabled the successful quantification of gadoteric acid in various environmental clay soil samples.

In peat, high background levels of endogenous Gd^3+^ necessitated an EDTA-based clean-up step to ensure reliable quantification during validation with fortified samples. The method demonstrated quantitative recovery and suitable precision for D in this matrix. The procedure, however, could not be extended to GB and GT due to excessive lower recoveries preventing its accurate quantification. The LOQ for D (4 ng g^−1^) was well below the concentrations found in the analysed peat samples, confirming the method’s applicability to the proposed objective.

Application of the validated method to environmental samples—including clay soils from municipal parks in Castelló (Spain) and peat from a controlled greenhouse irrigation experiment—confirmed the occurrence of gadoteric acid, the most frequently administered GBCA in the region. D was detected above the LOD in all soil samples and above the LOQ in one case. In peat, a clear accumulation trend was observed, with a positive correlation between the concentration of D in irrigation water and its levels in the peat matrix.

Positive findings of GBCAs in these matrices provide evidence of their persistence and accumulation under conditions associated with reclaimed or fortified water irrigation. These outcomes underscore the need for reliable, matrix-specific analytical methodologies to support comprehensive monitoring programs for GBCAs, recognized as emerging environmental contaminants. The analytical framework developed here contributes to a better understanding of the environmental fate of GBCAs and their potential transfer into the terrestrial food chain.

## Supplementary Information

Below is the link to the electronic supplementary material.

Supplementary file1 (DOCX 2.12 MB)

## Data Availability

Data are available from the authors by request.
