# Supplementary material for: Determination of selected gadolinium-based contrast agents in soil: method validation and application
Source: Anal Bioanal Chem. 2026 Feb 11;418(9):2607–17. doi: 10.1007/s00216-026-06389-2 (PMC13079464; doi:10.1007/s00216-026-06389-2)
Supplement: Supplementary file 1 — Supplementary file1 (DOCX 2.12 MB) [file 216_2026_6389_MOESM1_ESM.docx]

**Determination of Selected Gadolinium-Based Contrast Agents in Soil: Method Validation and Application**

A.F. Roig-Navarro*, F. Soria-Prieto, E. Pitarch, R. García-Cubedo

Environmental and Public Health Analytical Chemistry, Research Institute for Pesticides and Water. Universitat Jaume I, Castelló, Spain

**Supplementary Information**

**2. Materials and Methods.**

2.3 Validation procedure. Procedure for the EDTA-mediated stripping of endogenous Gd^3+^ from the peat matrix.

A 25 g aliquot of native peat was weighed into a 250 mL polyethylene (PE) container and treated with 100 mL of 0.5 M EDTA. The stripping process was conducted using a rotary agitator at 30 rpm for 1 h. Following this, the peat was rinsed twice with deionized water and allowed to air-dry in a fume hood. This procedure was performed in quadruplicate, and the resulting peat samples were subsequently pooled and homogenized to be used as the matrix for the validation procedure.

**3. Results and discussion**

3.2. Extraction optimisation

Table S1. Molecular structure of the selected GBCAs.

| **Compound** | **Molecular structure** | **Molecular weight** |
| --- | --- | --- |
| Gadoteric acid | 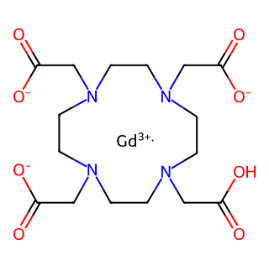 | 558.7 |
| Gadobutrol | 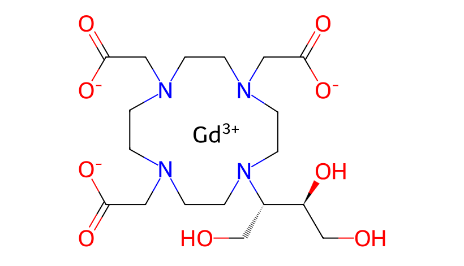 | 604.7 |
| Gadoteridol | 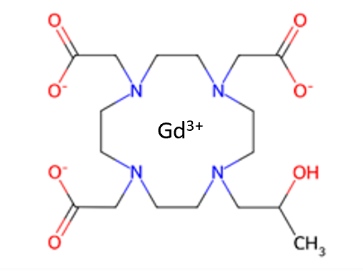 | 558.7 |

3.4. Application to real samples

Table S2. Concentration of the selected GBCAs found in several environmental water samples.

| Sample | Concentration (ng L^-1^) | | | | |
| --- | --- | --- | --- | --- | --- |
|  | Dotarem | Gadobutrol | | Gadobutrol | |
| WWTP_1_influent | 344 | 91 | | - | |
| WWTP_1_effluent | 276 | 115 | | - | |
| WWTP_2_influent | 122 | 17 | | - | |
| WWTP_2_effluent | 375 | 28 | | - | |
| WWTP_3_influent | 189 | - | | - | |
| WWTP_3_effluent | 54 | 3 | | - | |
| WWTP_4_influent | 500 | 46 | | - | |
| WWTP_4_effluent | 964 | 109 | | - | |
| Millars River. Upstream WWTPs | - | - | | - | |
| Millars River. Between WWTPs | 29 | 26 | | - | |
| Millars River. Downstream WWTPs | 30 | 3 | | - | |
| (-): below LOD |  | |  | |  |


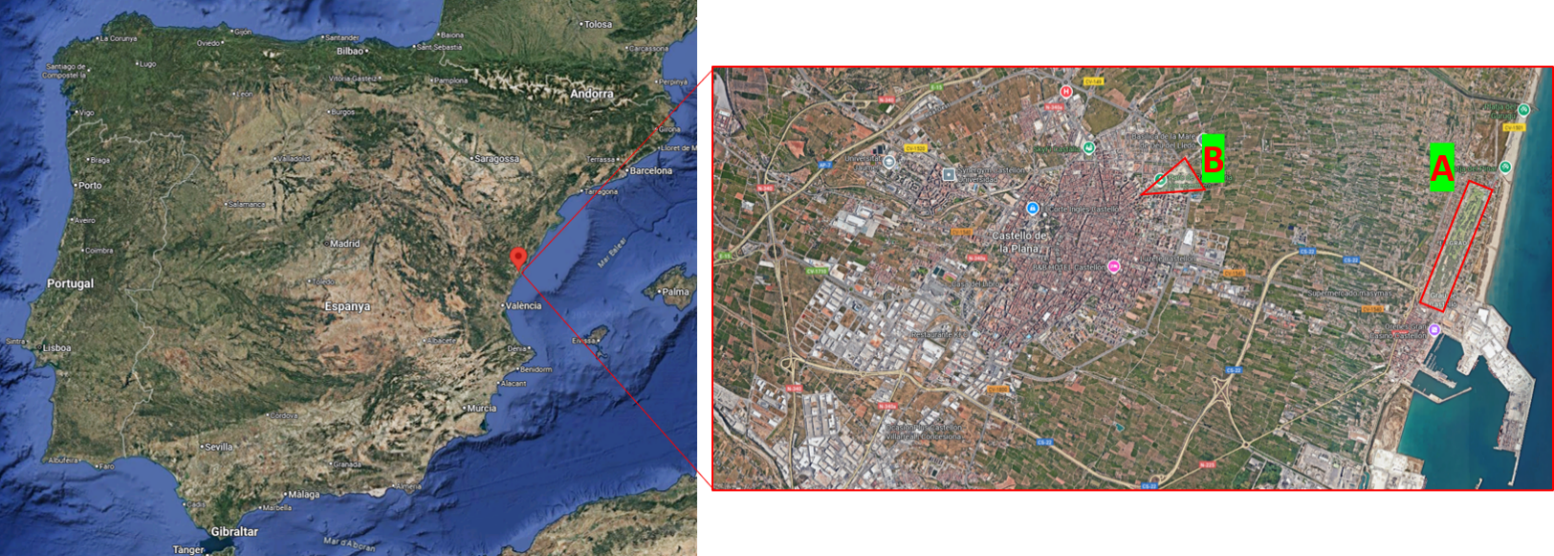


Figure S1. Castelló de la Plana municipal parks location: A) Litoral, B) Rafalafena


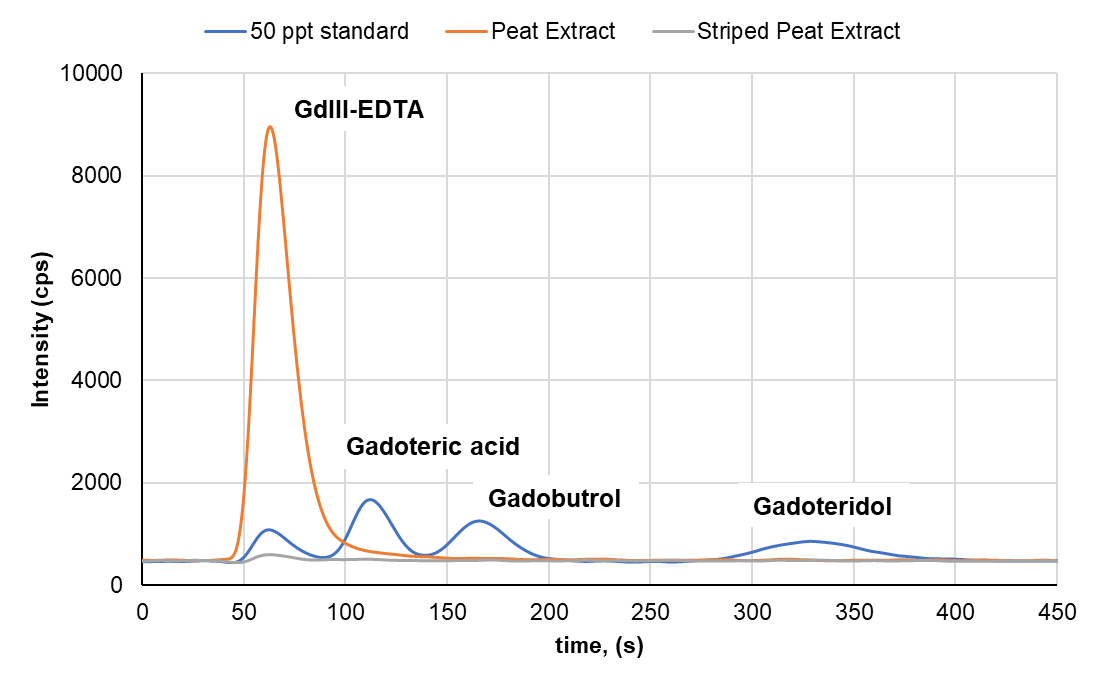


Figure S2. The overlaid chromatograms compare the peaks of a 50 ng L^-1^ GBCA standard solution with those of endogenous Gd^3+^ extracted from a peat sample, both before and after striping with EDTA.
